# Pretreatments of *Broussonetia papyrifera*: *in vitro* assessment on gas and methane production, fermentation characteristic, and methanogenic archaea profile

**DOI:** 10.5713/ajas.20.0503

**Published:** 2020-11-09

**Authors:** Lifeng Dong, Yanhua Gao, Xuelan Jing, Huiping Guo, Hongsen Zhang, Qi Lai, Qiyu Diao

**Affiliations:** 1 Feed Research Institute, Chinese Academy of Agricultural Sciences/Sino-US Joint Lab on Nutrition and Metabolism of Ruminant/CAAS-CIAT Joint Laboratory in Advanced Technologies for Sustainable Agriculture, Beijing 100081, China; 2 College of Life Science and Technology, Southwest Minzu University, Chengdu 610041, China; 3 College of Life Sciences, Henan Agricultural University, Zhengzhou 450002, China

**Keywords:** *Broussonetia Papyrifera*, Ensiling, *In vitro*, Methanogenic Archaea, Steam Explosion

## Abstract

**Objective:**

The present study was conducted to examine the gas production, fermentation characteristics, nutrient degradation, and methanogenic community composition of a rumen fluid culture with *Broussonetia papyrifera* (*B. papyrifera*) subjected to ensiling or steam explosion (SE) pretreatment.

**Methods:**

Fresh *B. papyrifera* was collected and pretreated by ensiling or SE, which was then fermented with ruminal fluids as ensiled *B. papyrifera* group, steam-exploded *B. papyrifera* group, and untreated *B. papyrifera* group. The gas and methane production, fermentation characteristics, nutrient degradation, and methanogenic community were determined during the fermentation.

**Results:**

Cumulative methane production was significantly improved with SE pretreatment compared with ensiled or untreated biomass accompanied with more volatile fatty acids production. After 72 h incubation, SE and ensiling pretreatments decreased the acid detergent fiber contents by 39.4% and 22.9%, and neutral detergent fiber contents by 10.6% and 47.2%, respectively. Changes of methanogenic diversity and abundance of methanogenic archaea corresponded to the variations in fermentation pattern and methane production.

**Conclusion:**

Compared with ensiling pretreatment, SE can be a promising technique for the efficient utilization of *B. papyrifera*, which would contribute to sustainable livestock production systems.

## INTRODUCTION

*Broussonetia papyrifera* (*B. papyrifera*), known as paper mulberry, is a deciduous tree of the Moraceae family, which is widely distributed in China, India, Thailand, and many other countries. The most revealing characteristic of *B. papyrifera* is its highly variable leaves, ranging from 7 to 20 cm in length. *B. papyrifera* has a high neutral detergent fiber (NDF) content, ranging 39.50 to 58.78 g/kg on a dry matter (DM) basis, which is close to that of sorghum (44.69%±2.02% of DM) [1]. It is reported that there are approximately over 5 million hectares of *B. papyrifera* in China and an abundance of *B. papyrifera* leaves and stalk resources is produced based on a large planting area every year [2]. However, application of *B. papyrifera* in livestock production system is restricted due to its high lignocellulosic contents and low nutritional values. Most of *B. papyrifera* residues are abandoned or disposed in the field casually, and some parts of them are burned by local famers causing a tremendous burden on the surrounding environment and lives of people [3]. Therefore, there is increasing interest to develop an ecological and sustainable way to use *B. papyrifera* to avoid the waste of natural resources and environment pollution.

Like other agricultural biomass materials, *B. papyrifera* contains high content of structural carbohydrates (cellulose, hemicellulose) and lignin. This recalcitrant nature of lignocellulosic biomass is the main limitation to biodegradability and biotransformation, hindering nutrient digestion and utilization by animals [4]. Previous research showed that agricultural residues such as corn stover, cotton stalk, and sorghum can be used as alternative feed resources when appropriate pretreatments were applied to break down the lignocellulosic structure [5]. Steam explosion (SE) is frequently used to process agricultural residues or crops for maximum lignocellulosic biomass digestibility or chemical product recovery [6,7]. This efficient and cost-effective approach involves heating biomass using saturated steam, followed by an explosive decompression of the reaction system to unlock the complex associations between cellulose, hemicellulose, and lignin [8,9]. A recent study showed that SE altered the physical and chemical structure of corn stover, leading to the improvement of the ruminal microbial colonization and degradation of cellulose and hemicellulose of dairy cows [7]. However, methane production would occur when the lignocellulosic biomass is being fermented in the rumen, leading to a huge proportion of dietary energy loss and contributing to the accumulation of greenhouse gases. Eom et al [10] reported that rubber wood waste pretreated with SE enhanced methane production from 10.9 to 83.9 L/kg volatile solid during anaerobic fermentation, while methane production from steam-exploded corn stover increased by 55% when compared with untreated materials [11]. Meanwhile, as one of the well-established methods to preserve green crops, ensiling is also widely used for efficient biomass degradability and minimum loss of nutrients in renewable energy production. Previous studies demonstrated that recalcitrance of the biomass can be alleviated during the ensiling process, and the improved availability of nutrients can increase methane production [12]. We thus hypothesized that SE and ensiling pretreatments of *B. papyrifera* could efficiently contribute to the degradation, and conversion of lignocellulosic biomass into volatile fatty acids (VFAs) production during anaerobic fermentation.

However, to the best of our knowledge, little literature is available on utilization of *B. papyrifera* for lignocellulose degradation and dynamic changes of methanogenic composition under different pretreatment methods. Therefore, the effects of ensiling and SE pretreatments on methane production, fermentation characteristics, and lignocellulosic degradation of *B. papyrifera* were evaluated. To further understand the role of methanogenic community in regulating gas production, functional *mcr*A gene sequencing technique was used to investigate the methanogenic dynamics, and the relationships between methanogen profile and fermentation performance.

## MATERIALS AND METHODS

### Raw material and inoculum

*B. papyrifera* were harvested on the 26th May 2019 from the experimental farm of Feed Research Institute of Chinese Academy of Agricultural Sciences (Zhoukou, Henan, China). These raw materials were harvested when the plant reached a height of 1.2 m and chopped into an approximate length of 10 cm with a manual forage chopper.

The inoculum used in this study was collected from six Dorper×thin-tailed Han crossbred sheep (mean±standard deviation; body weight, 36.2±2.45 kg; age, 25±2.7 months) fitted with rumen cannula. A basal diet containing Chinese wildrye (Leymus chinensis) hay and pelleted concentrate (forage to concentrate ratio = 50:50, DM basis; Supplementary Table S1) was formulated and provided at 07:00 and 19:00 *ad libitum* in amounts resulting in 5% refusals. Ruminal contents were collected from each sheep after the morning feeding (within 2 h). Ruminal samples were then mixed and filtered through four layers of cheesecloth into pre-warmed (39°C) thermos flasks that were filled with CO_2_ for anaerobic fermentation experiment. All animal procedures were reviewed and approved by the Animal Ethics Committee of the Chinese Academy of Agricultural Sciences (protocol number 057-2019).

### Pretreatments

#### Ensiling pretreatment

*B. papyrifera* was ensiled with *Lactobacillus plantarum* (10^6^ cfu/g of fresh weight) and packed into vacuum-sealed plastic bags (dimensions 225 mm×350 mm). A total of 10 bags were made and stored at ambient temperature for 45 days. Following ensiling, 4 bags were randomly opened and completely mixed for compositional analysis and anaerobic fermentation experiment. *Lactobacillus plantarum* used in the present study was purchased from Lallemand Inc. (Quebec, Canada), which was previously used in an earlier report of our group [13].

#### Steam explosion pretreatment

The SE pretreatment was performed using laboratory scale equipment (QB-200, Gentle bioenergy Co., Ltd., Henan, China) with a capacity of 2,500 g of samples per batch as previously described by Chang et al [14]. In order to obtain optimal SE conditions, a serial of preliminary experiments with different reaction pressures (1.2 and 1.4 Mpa), cooking times (60, 80, 100, 120, 140, 160 s), and temperatures (150°C and 194°C) were conducted. According to the recommendations of other researchers [7, 14], the optimal SE parameter of *B. papyrifera* was selected to 100 s under 1.2 Mpa with a temperature of 194°C. The steam-exploded *B. papyrifera* samples were stored in sealed plastic bags for the anaerobic fermentation experiment. In addition, a portion of *B. papyrifera* without any physical or chemical pretreatments was used as control treatment.

### Anaerobic fermentation

To assess the effects of different pretreatments on gas production of *B. papyrifera*, the anaerobic fermentation experiment was conducted with 16 bottles in each group: i) ensiled *B. papyrifera* (BPS) group, ii) steam-exploded *B. papyrifera* (BP-SE) group, and iii) untreated *B. papyrifera* (BP) group. A negative control was used with only buffered rumen fluid [15]. Fresh *B. papyrifera* or pretreated *B. papyrifera* were incubated together with a mixture of ruminal fluid and buffer solution (1:2) for 72 h. Buffer solution was formulated in line with the modified method of Hariadi et al [16] that included carbonate buffer, micromineral, and micromineral solution. The working volume of each bottle was 250 mL, and 30 mL inoculum (a mixture of ruminal fluid and buffer solution) was loaded into each bottle containing 500 mg of untreated or ensiled or steam exploded *B. papyrifera* as a substrate. These bottles were gently shaken before placing them in a thermostatically controlled shaking water bath at 39°C (shaking frequency, 40 r/min). At each incubation stage stated below, a small volume of gas was collected for determination of CH_4_ production. At the end of anaerobic fermentation, all the bottles were placed on ice to suppress the microbial growth and gas production.

### Sample collection and analysis

#### Gas production

Gas production was recorded before incubation (0 h) and after 1, 3, 6, 9, 12, 24, 36, 48, 60, and 72 h of fermentation. Four bottles were randomly selected from each treatment for determination of methane production at 12, 24, and 72 h of incubation using a gas chromatograph (GC-8A, Shimadzu Corp., Kyoto, Japan) with a flame-ionization detector. The temperature of steel capillary column (Porapack Q, 80/100 mesh, Waters Associates Inc., Milford, MA, USA) was maintained at 65°C and ultra-high purity nitrogen (99.999%) was used as the carrier gas at 40 mL/min flow.

#### Fermentation Characteristics and lignocellulosic degradability

The incubation fluid from each treatment was used for measurement of fermentation parameters. The pH of the incubation contents was determined using a digital pH meter (Hanna, Hi 8520, Ronchi di Villafranca, Italy). The VFA concentration and the molar VFA profile including acetate, propionate, butyrate, isobutyrate, valerate, and isovalerate was measured using GC (Agilent Technologies, Palo Alto, CA, USA) with a capillary column (BP21, 30 m×0.25 mm ID×0.25 μm; Kinesis Inc., Vernon Hills, IL, USA). Lignocellulosic degradation of *B. papyrifera* samples were determined using the Daisy II incubator system for 72 h of incubation (ANKOM Tech., Fairport, NY, USA). This reaction unit consisted of four incubation jars containing 1.6 L of buffer solution and 0.4 L of rumen liquor with 25 nylon bags inside. Buffer formulation was based on Maggiolino et al [17] study and included 1.33 L buffer A and 266 mL of buffer B. Before the commencement of the incubation, nylon F57 filter bags (ANKOM Tech., USA) were rinsed in acetone and dried at 100°C for 24 h. Reprehensive samples from three pretreatments were dried in an air-force oven at 65°C for 48 h and ground through a 1-mm screen (Wiley mill, A. H. Thomas, Philadelphia, PA, USA) for incubation. The percentage loss in weight was measured as *in vitro* degradability at 24 and 72 h.

#### Methanogenic community composition

Methanogenic community composition in the incubation fluids (at 6, 12, 24, and 72 h of incubation) was examined by using high-throughput sequencing technique targeting the functional gene of methyl-coenzyme M reductase A (*mcr*A). The total genomic deoxyribonucleic acid was extracted with a QIAamp Fast DNA Stool Mini Kit (QIAGEN, Valencia, CA, USA) according to the manufacturer’s instructions. The real-time polymerase chain reaction (PCR) primers used to amplify the mcrA fragments was designed according to the method of Luton et al [18]: 5′-GGTGGTGTMGGATTCACACAR TAYGCWACAGC-3′ and 5′-TTCATTGCRTAGTTWG GRTAGTT-3′. The whole reaction was performed by using the TaKaRa rTaq DNA Polymerase system, which included a 2 μL of 10×buffer, 2 μL of deoxynucleotide triphosphate (dNTPs) mixture (2.5 mmol/L), 0.2 μL of rTaq polymerase, and 0.8 μL of each primer (forward and reverse). The PCR amplification was initiated at 95°C for 3 min, followed by 30 cycles of 95°C for 30 s, 55°C for 30 s, and 72°C for 45 s, and finished with a final extension of 72°C for 10 mins by using a 2700 GeneAmp PCR system (Applied Biosystems, Foster, CA, USA). Amplicons were then extracted from 1% agarose gels and purified using the AxyPrep DNA Gel Extraction Kit (Axygen Biosciences, Union City, CA, USA) according to the manufacturer’s instructions and were quantified using QuantiFluor-ST (Promega, Madison, WI, USA). Library was quantified and analyzed using the Agilent 2100 Bioanalyzer and the ABI StepOnePlus Real-Time PCR system. The qualified libraries were then sequenced on the Illumina HiSeq platform according to standard protocols. The raw sequence data were processed, and the quality filtered sequence were clustered into operational taxonomic units (OTUs) according to the 97% sequence similarity using QIIME.

### Statistical analyses

Data processing and statistical analysis were performed using MIXED procedure in SPSS (version 22.0 for Windows, SPSS Inc., Chicago, IL, USA). Data on gas production and fermentation parameters (including pH, VFA, NH_3_) were the average of quadruplicate results within incubation. Based on the taxonomic assignments, the relative abundance of methanogenic archaea was determined at the genus level (expressed as percentages). Archaeal population diversity, evenness, and richness values were determined by using QIIME software (version 17.0). These data were analyzed using Welch’s t-test package and were considered statistically different at p<0.05. Paleontological statistics program was used to develop the relationship between methanogenic archaea and fermentation profiles and methane production. The Spearman correlation coefficient was calculated, and the correlation matrix was visualized in heatmap format

## RESULTS AND DISCUSSION

### Chemical composition of untreated and ensiled or steam-exploded *Broussonetia papyrifera*

The chemical composition of the untreated raw material, *B. papyrifera* silage, and steam-exploded *B. papyrifera* used in the present study is shown in Table 1. Compared with the untreated samples, the NDF content of *B. papyrifera* silage and steam-exploded *B. papyrifera* decreased by 35% and 14%, respectively. These results are similar to the studies of Zhao et al [7] and Chang et al [14] who reported that the NDF content of ensiled rice straw and steam-exploded corn stover decreased by 9.58% and 30.06%, respectively, indicating that steam-explosion pretreatment was more effective in breaking down the composition of lignocellulose biomass. It is reasonable that the lignocellulosic structure is more extensively deconstructed since SE involves elevated temperature heating and a rapid release of pressure in a short time. Correspondingly, *B. papyrifera* subjected to ensiling or steam-explosion pretreatment had high calculated NFC values of 30.8% and 36.1%, respectively, making the *B. papyrifera* biomass a promising material for renewable feedstock and bioenergy production.

### Effects of pretreatments on cumulative gas and methane production of *Broussonetia papyrifera*

**Figure 1:**
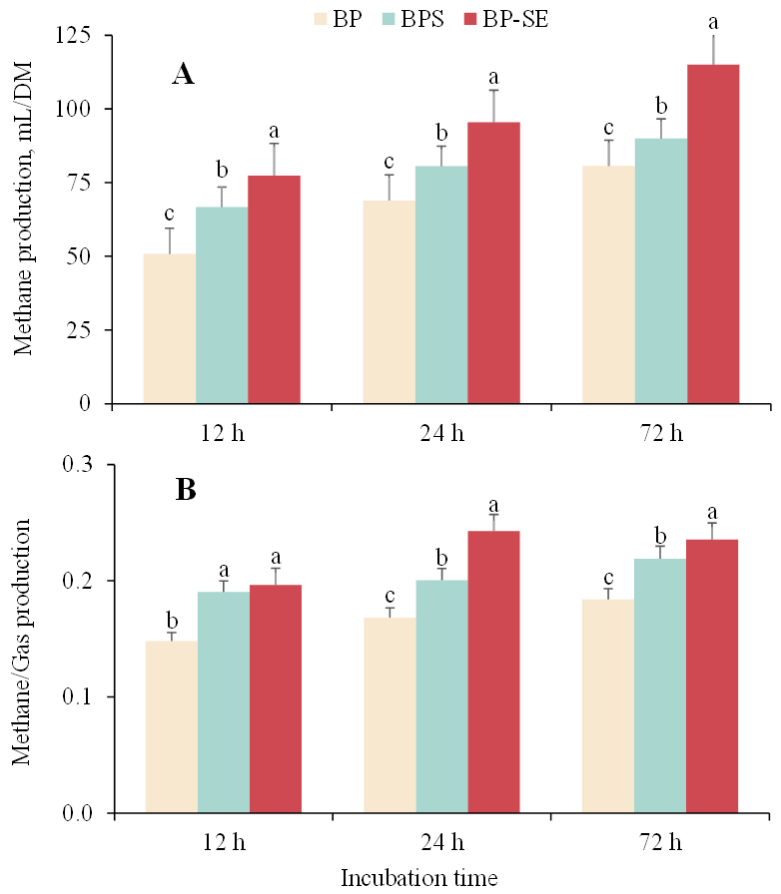
Variations in methane production (A) and methane production as a proportion of total gas production (B) from *Broussonetia papyrifera* (BP), *Broussonetia papyrifera* silage (BPS), and steam-exploded *Broussonetia papyrifera* (BP-SE) pretreatments when incubated for 12, 24, and 72 h. ^a–c^ Different letters in each figure indicate significant difference (p<0.05).

The cumulative gas and methane production are shown in Table 2 and Figure 1. Overall, the cumulative gas produced in the first 48 h increased significantly but reached asymptotic conditions at 72 h. Compared with the gas produced from untreated material (193.0 mL/DM), the total gas production from *B. papyrifera* silage and steam-exploded *B. papyrifera* was 223.6 and 241.6 mL/DM, respectively (p<0.05). The amount of methane gas production from untreated, ensiled or steam-treated *B. papyrifera* were 80.7, 89.9, and 115.1 mL/DM, respectively (Figure 1A). Methane yield as a proportion of total biogas production from steam-exploded pretreatment was 30.9% and 17.7% higher than untreated and *B. papyrifera* silage when incubated for 24 h (Figure 1B). As gas and methane production is associated with the chemical composition of the biomass, SE pretreatment contributes to the hemicellulose degradation and lignin transformation, and consequently, reduces biomass recalcitrance, and produces higher gas yield [8,11]. Liu et al [12] reported that increased gas and methane values had resulted from the production of large amount of organic acids during the ensiling process; on the other hand, and a good relationship was found between methane production and the concentration of acetic acid [19].

The results obtained in the present study indicated that both ensiling and steam-explosion pretreatments can contribute to the disruption of lignocellulosic structures, resulting in increase in gas production although the mechanism of structural carbohydrate degradation varied. Due to the continuous fiber degradation and methane production, the methane/gas production for BP-SE measured at 24 h was close to that value when measured at 72 h. In addition, we observed the highest methane content (23%) when steam-exploded *B. papyrifera* was fermented for 24 h, which was higher than that reported by Zhang et al [20] who found that VFA accumulation from the rumen fluid and the biomass acidification might inhibit methanogenic activity and methane yield. On the other hand, the inhibition of methane production can be attributed to the limited fermentable substrate during the anaerobic incubation as more content of degradable fiber would result in a greater methane production [15,21]. However, although increased methane production resulted from with structural carbohydrate degradation of steam-exploded *B. papyrifera* fermented with ruminal fluid, it would be not favorable when *B. papyrifera* is included in the diet of ruminants as methane is one of the greenhouse gases and represents a source of energy loss to the animal production. More research may be needed to determine the appropriate levels of steam-exploded *B. papyrifera* supplemented in the ruminant diet to achieve the balance of efficient degradation of lignocellulosic biomass and production of enteric methane emissions.

### Effects of pretreatments on fermentation characteristics and lignocellulosic degradability of *Broussonetia papyrifera*

**Figure 2:**
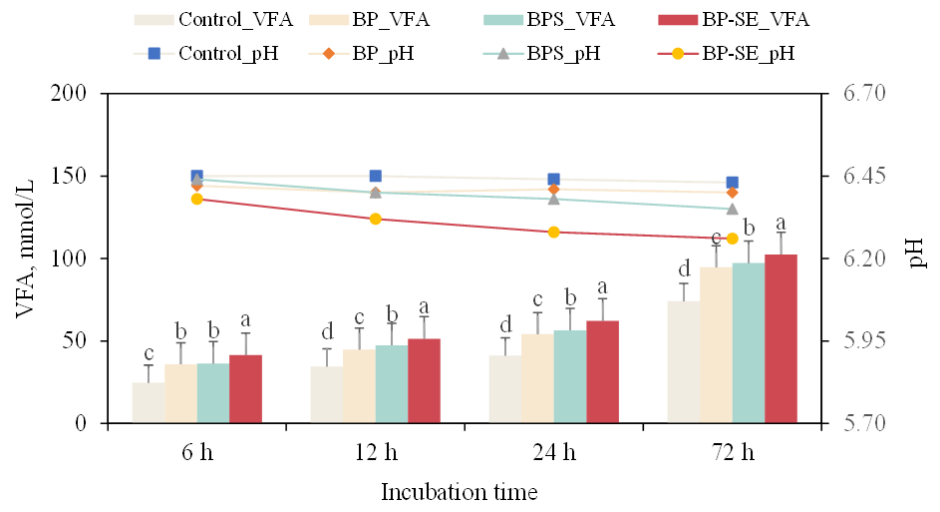
Variations in volatile fatty acid (VFA) concentration and pH from *Broussonetia papyrifera* (BP), *Broussonetia papyrifera* silage (BPS), and steam-exploded *Broussonetia papyrifera* (BP-SE) pretreatments when incubated for 6, 12, 24, and 72 h. ^a–d^ Different letters in each figure indicate significant difference (p<0.05).

Total VFA production and pH values, and individual VFA production are shown in Figure 2 and Table 3, respectively. Overall, an opposite tendency of pH and VFA concentration was observed during the whole process of incubation, which agrees with other reports of *in vitro* experiments [15,20]. The pH values can indirectly reflect the anaerobic incubation condition, and closely correlate with VFA production, buffer capacity, and activity of acidogenic bacteria [20]. In the present study, the lowest pH value of 6.26 was observed for steam-treated *B. papyrifera* at the end of incubation, which corresponds to the maximum VFA concentration of 102.43 mmol/L. Zhou et al [15] suggested that elevated pH values in the rumen would suppress the fermentation process, which would result in less VFA production and consequently decreased acetate:propionate ratios. However, the lowest pH values observed during the incubation were within the optimal range, which would be beneficial for microbial activities and cellulose digestion [22,23].

Lignocellulosic biomass is more easily fermented to acetate and hydrogen than propionate [20]. In the present study, acetate was the dominant component of VFA throughout the whole incubation process, which respectively increased from 20.8 to 56.2 mmol/L and from 23.9 to 60.4 mmol/L for ensiled and steam-exploded *B. papyrifera* after 72 h of incubation. Increased acetate and butyrate and decreased propionate are closely related to enhanced methane production. Highest acetate values and reduced acetate:propionate ratio for steam-exploded *B. papyrifera* observed in the present study demonstrated that SE pretreatment can effectively remove the cellulosic links and contribute to the formation of acetate and butyrate. This would consequently contribute to a greater fiber degradation and ruminal reduction of CO_2_ into methane by methanogens [16].

**Figure 3:**
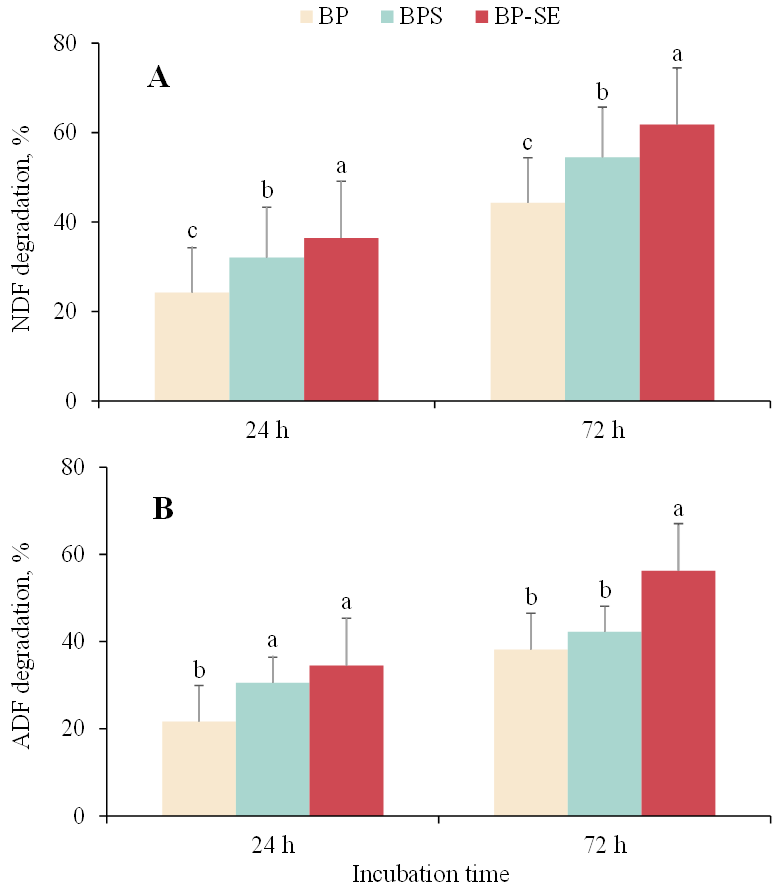
Variations in neutral detergent fiber (NDF) and acid detergent fiber (ADF) degradation when *Broussonetia papyrifera* (BP), *Broussonetia papyrifera* silage (BPS), and steam-exploded *Broussonetia papyrifera* (BP-SE) pretreatments when incubated for 24 and 72 h. ^a–c^ Different letters in each figure indicate significant difference (p<0.05).

As shown in Figure 3A, significant improvement of NDF degradation was observed for ensiled or steam-exploded *B. papyrifera* when compared with untreated material either at 24 or at 72 h. Acid detergent fiber (ADF) of steam-exploded *B. papyrifera* was also degraded to a significant extent after 72 h of incubation (p<0.05), whereas similar ADF degradation values were observed between untreated or ensiled *B. papyrifera* (p>0.05). Zhao et al [7] found a significant improvement of degradation of steam-exploded corn stover compared to untreated material, which was in agreement with the results of Shi et al [24] that the degradation of neutral detergent solute was significantly increased by 25.1% after SE treatment. Similarly, previous study reported that enhancement of fiber degradation and disruption of the crystalline structure of lignocellulose biomass occurred during the ensiling process. The breakage of the bond among cellulose, hemicellulose, and lignin provides the opportunity for the colonization of ruminal microbiota and excretion of fiber-degrading enzymes [25]. Furthermore, methane production is well known to be related to the anaerobic degradation of lignocellulose biomass. The increased degradation of NDF and ADF recorded in this study was in accordance with enhanced methane production for ensiled or steam-exploded *B. papyrifera*, suggesting that pretreatments could help the activity of ruminal microorganisms and achieve greater biogas yield from *B. papyrifera*.

### Effects of pretreatments on methanogenic archaea profile of *Broussonetia papyrifera*

#### Methanogenic diversity and richness

**Figure 4:**
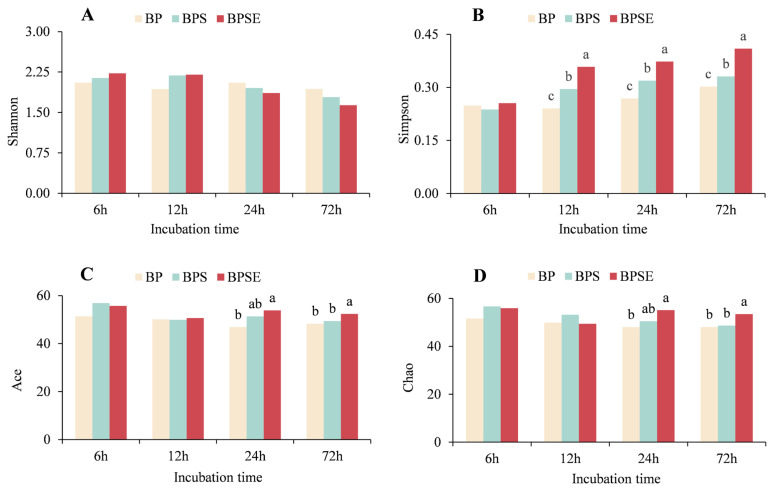
Methanogenic diversity from *Broussonetia papyrifera* (BP), *Broussonetia papyrifera* silage (BPS), and steam-exploded *Broussonetia papyrifera* (BPSE) pretreatments when incubated for 6, 12, 24, and 72 h. ^a–c^ Different letters in each figure indicate significant difference (p<0.05).

After sequencing analysis and data processing, the average number of valid sequences was 18,755±3,178.5, 20,220±4,346.8, 17,298±4,102.6, and 18,991±3,657.5 for each incubation time (6, 12, 24, and 72 h), respectively. The Good’s coverage over 0.999 of all samples indicated that most common OTUs were identified and included into the analysis. Richness and diversity indices of methanogenic communities are shown in Figure 4. The Shannon indices did not change significantly among the three pretreatments throughout the incubation, whereas ensiled or steam-exploded pretreatment showed higher Simpson values than untreated material when incubated for 12, 24, or 72 h (p<0.05). In addition, steam-exploded pretreatment significantly enhanced the richness of the methanogenic community as Ace and Chao indices were highest among the three treatments at 24 and 72 h (p<0.05). These results demonstrated that ensiled or steam-exploded pretreatment enhanced diversity and richness of the methanogenic community during the incubation. Li et al [11] suggested that the improved biodiversity may be ascribed to the positive effects of methanogenesis and more accessible substrates for the growth of anaerobic microbes and methanogens. This conclusion was in line with our findings that increased VFA production was observed for ensiled or steam-exploded of *B. papyrifera*, which was beneficial to the substance degradation and high incubation performance.

#### Methanogenic community composition

**Figure 5:**
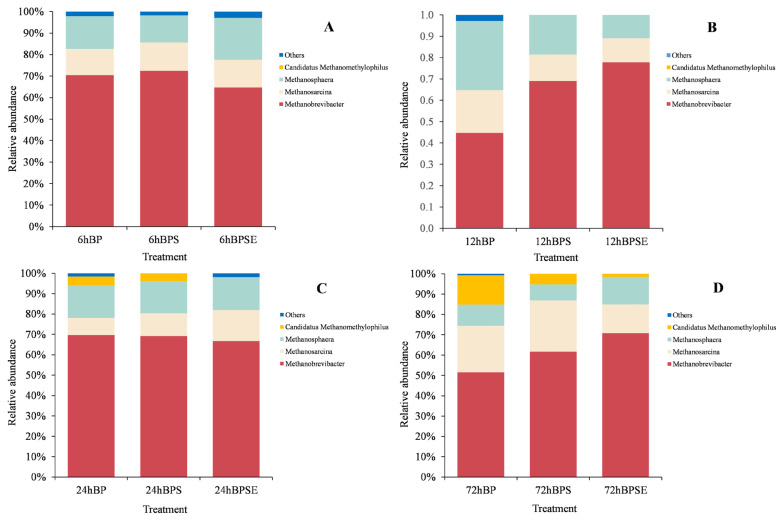
Relative abundance of the methanogenic composition at genus levels from *Broussonetia papyrifera* (BP), *Broussonetia papyrifera* silage (BPS), and steam-exploded *Broussonetia papyrifera* (BPSE) pretreatments when incubated for 6, 12, 24, and 72 h.

Taxonomy analysis of methanogenic communities at the genus level is shown in Figure 5. Three major genera of *Methanobrevibacter*, *Methanosarcina*, and *Methanosphaera* were identified within the whole archaeal sequences in all samples. *Methanobrevibacter* was the most dominant genera with a relative abundance of 68.9%, 63.9%, 67.1%, and 59.4% when incubated for 6, 12, 24, and 72 h, respectively. This result was similar to the findings from Dong et al [26] who reported *Methanobrevibacter* accounted for 48.4% to 56.4% among methanogenic genera in ruminal samples. It was reported that sequences belonging to *Methanobrevibacter* accounted for approximately 62% of rumen archaea, with most of sequences relating to *Methanobrevibacter gottschalikii* (33.6%) and *Methanobrevibacter ruminantium* (*M. ruminantium*; 27.3%). In conformance with previous results, *Methanobrevibacter* sp. SM9 and *M. ruminantium* species were identified in the present study using the functional gene of *mcr*A sequencing technique, which accounted for 38.6% and 17.8%, respectively. *Methanosarcina* population was the second most abundant methanogen at each incubation period and was reported to be a multifunctional methanogen that could utilize either hydrogen/carbon dioxide or acetate for methane production [8,12]. Theuretzbacher et al [9] reported that increased abundance of hydrogen-utilizing methanogens during fermentation was associated with high levels of dissolved organic matter. It was also reported that increased abundance of *Methanosphaera* represented enhanced consumption of hydrogen, which would consequently reduce the H_2_ accumulation and contribute to conversion of lignocellulose biomass to VFA [12].

The methanogen community structure was dynamic among the three treatments (Figure 5). Steam-exploded pretreatment consistently showed a higher population of *Methanobrevibacter* during the incubation but relatively low abundance of *Methanosarcina* when compared with ensiled pretreatment. The order of *Methanosarcinales* (e.g., *Methanosarcina barkeri* and *Methanosarcina mazei*) can use methanol and methylamines as substrates, but also metabolize acetate for methanogenesis. However, although both genera contributed to the methane generation, the metabolism activity and substrate utilization and conversion is still unclear. In addition, a small proportion of *Candidatus Methanomethylophilus* was observed among the three pretreatments when incubated for 24 and 72 h. Dong et al [26] reported a low abundance of *Candidatus Methanomethylophilus* (5.8%) in ruminal fluid and found that it was correlated with propionate concentration.

#### Correlation analysis of the methanogenic profile and fermentation parameters

**Figure 6:**
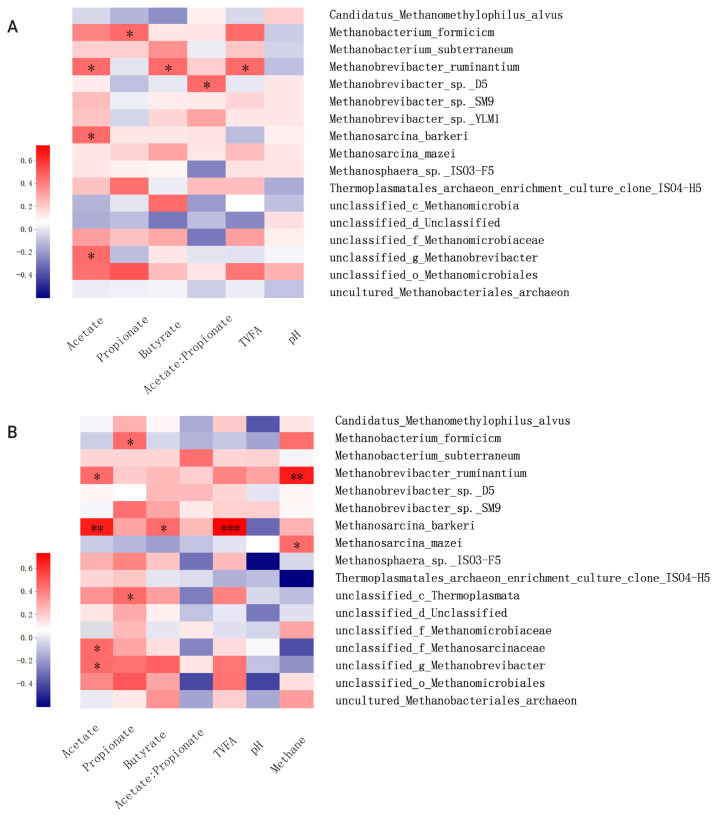
Correlation analysis of the methanogenic archaea and fermentation profiles when incubated for 6 and 24 h (A and B).

Methane formation is the final product of methanogenic archaea that use hydrogen or other substrates during the anaerobic fermentation of lignocellulosic biomass. Meanwhile, production and accumulation of VFA during incubation can reflect microbial activities and methanogenesis function. In the present study, the correlation matrix between the relative abundance of methanogen and fermentation parameters and methane production were examined by Spearman’s correlation heatmap at the species level (Figure 6). The correlation analysis showed that *M. ruminantium* was positively correlated with acetate, butyrate, total VFA concentration, and methane production when incubated at 6 or 24 h. Previous studies showed that *Methanobrevibacter* is the most abundant rumen archaea, with sequences mainly being associated with *M. ruminantium* (28%) and *M. gottschalkii* (33.6%) [27]. As the main hydrogen-utilizing methanogen, the abundance and activity of *M. ruminantium* is significantly affected by the production of acetate and butyrate, which is accompanied by the hydrogen formation during incubation. In addition, based on the complete genomic sequence analysis of *M. ruminantium* conducted by Leahy et al [27] it was suggested that *M. ruminantium* may be one of the protozoa-associated methanogens, and it can effectively obtain hydrogen for methanogenesis through hydrogen transfer from protozoa [26]. Meanwhile, positive correlation was also observed between butyrate concentration and *Methanobrevibacter* sp. D5 and *Methanobrevibacter* sp. *SM9*, which may be explained by the similar methanogenesis pathway and central metabolism to *M. ruminantium*. *Methanosphaera* sp. *ISO3-F5* was reported at a low relative abundance accounting for an average abundance of 8.2%±6.7% in the rumen of cattle and sheep [26]. As one of the main methylotrophic methanogens, *Methanosphaera* sp. *ISO3-F5* can utilize hydrogen to convert methanol into methane instead of reducing carbon dioxide to methane. In the present study, acetate was positively correlated with *Methanosphaera* sp. *ISO3-F5*, which would result from the increased availability of hydrogen when acetate formed during lignocellulosic degradation. Moreover, acetate concentration was positively related to the abundance of *Methanosarcina barkeri* and *Methanosarcina Mazeiin* in the present study, which was in agreement with the previous studies that acetate and other fermentation products (e.g., methanol and methylamines) were main substrates for the methane production by these two methanogen strains [24]. In conclusion, this study demonstrated that ensiling and SE pretreatments have a great potential for effective degradation of *B. papyrifera*. Compared with ensiling pretreatment, SE pretreatment decreased the ADF and NDF contents by 39.4% and 10.6%, respectively, after 72 h incubation. These results were closely related to the dynamic variations of methanogenic diversity and relative abundance. It is concluded that SE pretreatment may be a promising technique for the efficient utilization of *B. papyrifera*, contributing to sustainable livestock production systems

## Figures and Tables

**Table 1:** Chemical composition of *Broussonetia papyrifera*, *Broussonetia papyrifera* silage, and steam-exploded *Broussonetia papyrifera*

Item^1)^	OM	CP	EE	NDF	ADF	Ash	NFC^1)^

	----------------------------------------------- g/kg DM basis --------------------------------------------						
*Broussonetia papyrifera*	913	143	49.1	389	198	86.6	333
*Broussonetia papyrifera silage*	865	158	48.4	251	171	134.9	408
Steam-exploded *Broussonetia papyrifera*	894	188	61.2	334	260	106.1	311

DM, dry matter; OM, organic matter; CP, crude protein; EE, ether extract; NDF, neutral detergent fibre; ADF, acid detergent fibre; NFC, non-fibrous carbohydrate.

1) NFC is calculated as 100–(NDF+CP+EE+ash).

**Table 2:** Gas production from untreated, ensilage and steam-exploded Broussonetia papyrifera at different incubation times

Item	Gas production (mL/DM)									

	1 h	3 h	6 h	9 h	12 h	24 h	36 h	48 h	60 h	72 h
BP^1)^	17.0^b^	40.7^c^	93.3^b^	123.5^b^	143.1^c^	158.6^c^	172.4^c^	183.0^c^	188.3^c^	193.0^c^
BPS^1)^	18.2^b^	46.0^b^	97.7^a^	130.3^a^	150.6^b^	177.0^b^	204.1^b^	209.3^b^	222.1^b^	223.6^b^
BP-SE^1)^	34.7^a^	65.5^a^	100.2^a^	132.8^a^	164.3^a^	197.0^a^	220.8^a^	231.1^a^	241.6^a^	241.8^a^
SEM	3.02	3.75	2.09	2.98	3.94	6.06	7.62	7.51	8.43	7.78
p-value	0.001	0.007	0.046	0.039	0.016	0.005	0.002	0.003	0.003	0.001

DM, dry matter; SEM, standard error of means.

1) BP, *Broussonetia papyrifera*; BPS, *Broussonetia papyrifera* silage; BP-SE, steam-exploded *Broussonetia papyrifera*.

a–c Means within a column with different superscripts differ significantly (p<0.05).

**Table 3:** Fermentation parameters of untreated, ensiled and steam-exploded *Broussonetia papyrifera* at various incubation times

Time	Treatments^1)^	Individual VFA production						

		Acetate^2)^	Propionate^2)^	Butyrate^2)^	Isobutyrate^2)^	Valerate^2)^	Isovalerate^2)^	Acetate/propionate
6 h	Control	0.549^c^	0.178^c^	0.098^c^	0.019	0.028	0.046^a^	3.08^a^
	BP	0.566^b^	0.220^b^	0.120^a^	0.021	0.024	0.049^a^	2.57^c^
	BPS	0.571^b^	0.213^b^	0.127^a^	0.019	0.025	0.045^a^	2.69^b^
	BP-SE	0.577^a^	0.226^a^	0.112^b^	0.021	0.023	0.041^b^	2.50^d^
	SEM	0.0156	0.0112	0.0050	0.0036	0.0018	0.0035	0.122
	p-value	0.011	<0.01	0.017	0.068	0.055	<0.01	<0.01
12 h	Control	0.553^c^	0.182^b^	0.098^c^	0.020^c^	0.030	0.047^a^	3.04^a^
	BP	0.555^c^	0.218^a^	0.120^a^	0.024^b^	0.034	0.049^a^	2.56^b^
	BPS	0.588^b^	0.189^b^	0.120^a^	0.025^b^	0.032	0.046^a^	3.11^a^
	BP-SE	0.593^a^	0.207^a^	0.111^b^	0.028^a^	0.020	0.041^b^	2.22^c^
	SEM	0.0133	0.0160	0.0113	0.0012	0.0015	0.0027	0.10^4^
	p-value	0.036	0.019	0.023	0.046	0.072	0.029	0.017
24 h	Control	0.549^c^	0.203^b^	0.100^b^	0.037^a^	0.042	0.049^a^	2.90^a^
	BP	0.579^b^	0.202^b^	0.113^a^	0.025^b^	0.034	0.047^a^	2.86^b^
	BPS	0.559^c^	0.194^c^	0.114^a^	0.028^b^	0.036	0.049^a^	2.89^a^
	BP-SE	0.595^a^	0.213^a^	0.114^a^	0.023^c^	0.038	0.037^b^	2.73^c^
	SEM	0.0180	0.0029	0.0071	0.0017	0.0029	0.0035	0.105
	p-value	0.032	0.014	0.787	<0.01	0.056	<0.01	0.021
72 h	Control	0.547^d^	0.205^c^	0.108^c^	0.030	0.039	0.051	2.67^b^
	BP	0.559^c^	0.213^b^	0.124^a^	0.027	0.040	0.037	2.63^b^
	BPS	0.578^b^	0.192^d^	0.116^b^	0.030	0.043	0.041	3.01^a^
	BP-SE	0.590^a^	0.228^a^	0.125^a^	0.026	0.044	0.040	2.55^c^
	SEM	0.0148	0.0079	0.0042	0.0018	0.0025	0.0034	0.098
	p-value	0.023	0.019	0.051	0.074	0.055	0.062	<0.01

SEM, standard error of means; VFA, individual volatile fatty acid is expressed as a proportion of VFA.

1) Control, only ruminal fluid and buffer; BP, *Broussonetia papyrifera*; BPS, *Broussonetia papyrifera* silage; BP-SE, steam-exploded *Broussonetia papyrifera*.

2) Unit for acetate, propionate, butyrate, isobutyrate, valerate, and isovalerate is mmol/L.

a–d Means within a column at the same time with different superscripts differ significantly (p<0.05).

## SUPPLEMENTARY MATERIAL

Supplementary Suppl S1. Ingredient and chemical composition (g/kg of DM, unless otherwise stated) of diet fed to sheep
