# Supplementary material for: Pretreatments of Broussonetia papyrifera: in vitro assessment on gas and methane production, fermentation characteristic, and methanogenic archaea profile
Source: Anim Biosci. 2020 Nov 9;35(9):1367–78. doi: 10.5713/ajas.20.0503 (PMC9449391; doi:10.5713/ajas.20.0503)
Supplement: Supplementary Suppl S1. — Ingredient and chemical composition (g/kg of DM, unless otherwise stated) of diet fed to sheep [file ajas-20-0503-suppl.pdf]

Supplementary Table S1. Ingredient and chemical composition (g/kg of DM, unless otherwise stated) of diet fed to sheep

Items[1)]	Content
Ingredients	
Chinese wildrye	50.0
Corn	32.9
Soybean meal	14.1
$CaHPO_4$	0.6
Limestone	1.0
NaCl	0.5
Premix	1.0
Total	100
Nutrient level	
DM, g/kg of fresh matter	376
OM	916
CP	86
EE	27
NDF	397
ADF	159
Ca	12
P	4
NFC	407

[1)] The premix provided the following per kg of diets: VA 15000IU, VD 2200 IU, VE 50IU, Fe 55 mg, Cu 12.5 mg, Mn 47 mg, Zn 24 mg, Se 0.5 mg, I 0.5 mg, and Co 0.1 mg.

NFC is a calculated value, NFC = 1 - (NDF + CP + EE + Ash); all values expressed as g/kg.
